# Which type of tourism short video content inspires potential tourists to travel

**DOI:** 10.3389/fpsyg.2023.1086516

**Published:** 2023-03-01

**Authors:** Guihua Wu, Xinyi Ding

**Affiliations:** College of Tourism, Huaqiao University, Quanzhou, China

**Keywords:** tourism short video, tourism content marketing, customer inspiration, travel intention, consumption orientation

## Abstract

While user-generated short videos have become very common in tourism marketing, how they affect potential tourists’ decisions has not been discussed academically. Based on the customer inspiration theory, this study explored the effects of different tourism short video contents on potential tourists’ travel intentions, as well as the mediating effect of customer inspiration and the moderating effect of consumption orientation through three experiments. The following conclusions were drawn. (1) Tourism short videos significantly increased potential tourists’ customer inspiration and travel intention; (2) The customer inspirations (“inspired-by” and “inspired-to”) chain-mediated the relationship between tourism short videos and potential tourists’ travel intentions; (3) Consumption orientation positively moderated the chained mediation effect above, and the chained mediation effect of tourism short videos on the travel intentions of tourists with hedonistic motivations through inspire-by and inspire-to is stronger than that of tourists with utilitarian motivations. The above findings could help expand the perspective of tourism short video research and provide suggestions for tourism business managers to apply short video content to marketing.

## 1. Introduction

With the popularization of smart mobile devices and the deployment of 4G networks, information technology advances have made self-expression through social media increasingly easier, which gives rise to user-generated tourism short videos. Short videos are those with durations under 5 min that can be shot, edited, and beautified quickly by smart mobile devices and shared in real-time on social media platforms (Cao et al., 2021; Chi et al., 2022). Tourism is one of the important areas of short video content production and dissemination, where travel itineraries, scenic environments, travel tips, etc., can be presented (Liao et al., 2020). Compared to other emerging social platforms such as microblogging and live streaming, short videos have a large user base (according to CNNIC, as of June 2022, the number of independent short video platform users in China has reached 962 million) and can vividly express the image of the destination and affect the tourists’ behavior intentions (Chi et al., 2022). As a result, short video platforms are becoming the preferred channel for tourism enterprises to market their products and a link for interaction, co-creation, and sharing between destinations and tourists (Wengel et al., 2022). However, due to the lack of management and guidance during production, a large number of short videos have extremely limited scope and frequency of distribution (Mulier et al., 2021; Wang et al., 2022). According to the 2022 TikTok Travel Ecology Report, the number of users with travel interests on TikTok exceeded 270 million in 2021, and over 79 million people published travel-related videos on the TikTok platform. Users with over 10,000 followers are only 30,000. The data show that nearly 99.6% of short video is not widely distributed and participants account for only 29.2% of those interested, indicating that the huge tourism short video marketing inputs have not yet been transformed into the travel behaviors of potential tourists. Therefore, effectively converting tourists’ attention to short videos into travel intentions or actual behaviors remains a practical problem requiring urgent solutions in the tourism industry.

Tourists usually conduct an information search before deciding to visit a destination (Gursoy, 2019). In the age of social media, short video social marketing has gradually replaced traditional information styles (such as touring paper brochures and static web images) and become the dominant information style with viral marketing (Eckler and Bolls, 2011). Considering the diverse forms of actual tourism short video content, it is a matter of concern whether the effects of different contents differ in the information search stage of potential tourists. Studies have shown that media content presentation is closely related to customer inspiration (Thrash and Elliot, 2004; Böttger et al., 2017). Derived from the general concept of “inspiration,” customer inspiration describes the activation of the customer from “inspired by external factors” to “inspired to practice new ideas” (Böttger et al., 2017). The concept of customer inspiration links the state of activation and acceptance of new ideas to the pursuit of consumption-related goals, thus could help in investigating the effects of short video marketing, i.e., how to, to a certain extent, increase customers’ consumption intentions while providing them with acceptable and even actively sought after contents in the current context of a fast-paced lifestyle, non-stop purchasing patterns, and the ever-shortening customer journey (Kuehnl et al., 2019). Therefore, customer inspiration can play an important role in the relationship between short video content and customers’ behavioral intentions.

Research has found that visual information could help to improve the fluency of information processing, resulting in more positive evaluation results (Mulier et al., 2021). Short videos that convey richer information and stimulate multi-sensory experiences are believed to have better persuasive effects in changing customer attitudes and behaviors (Tussyadiah and Fesenmaier, 2009). Although more and more scholars are interested in short video content marketing, existing studies focus on analyzing the impact effects brought by short video applications. For example, studies have explained the psychological mechanisms between short videos and willingness to travel from different theoretical perspectives, such as consumer trust, social relationships, and sense of presence (Liao et al., 2020; Cao et al., 2021; Chi et al., 2022). However, the above study lacks an analysis of the impact effect of tourism short videos from the perspective of their content characteristics. Although the above studies analyzed the effects of short videos, the complex process in which individuals produce new ideas and behavioral responses after receiving marketing guidance has been neglected. According to the customer inspiration theory, customer inspiration triggers positive emotions that lead to engagements in marketing activities, such as impulse purchases, product searches, and other positive responses (Thrash and Elliot, 2003). As a new marketing channel for tourism destinations, social media has become an important source of inspiration to drive tourist behaviors (Gao et al., 2021). Therefore, this study focused on the effect mechanism of tourism short videos on tourists’ behavioral intentions from a customer inspiration perspective.

In addition to external factors (e.g., short video content), tourists’ behavioral intentions are also influenced by their individual traits (e.g., self-esteem and motivation). Among them, travel intention is one of the most important prerequisites for participation in travel activities (Prebensen et al., 2013). According to the consumption orientation theory, customers generally conduct consumption activities based on utilitarian or hedonistic motivations (Hirschman and Holbrook, 1982; Silaban et al., 2022). In short video marketing, consumption orientations, especially hedonistic motivations, are important subjective conditions driving tourist behavior and providing direct intrinsic motivations for producing and maintaining travel behaviors (Pestana et al., 2020). Thus, customers with hedonistic motivations may be more easily motivated by emotional content (e.g., fun, novelty, and surprise) in short videos, which, in turn, may lead to stronger travel intentions. Therefore, this study further examines the moderating effect of consumption orientation between tourism short videos and potential tourists’ travel intentions.

Despite the insights from the existing literature (Böttger et al., 2017; Chi et al., 2022; Xue et al., 2022), the exact action mechanism by which tourism short video contents affect potential travelers’ travel intention has not been fully explored. To fill this research gap, this study designed three experiments in an attempt to reveal the effects of different tourism short video content on potential tourists’ travel intentions from the customer inspiration perspective. Specifically, Experiment 1 examined the effects of different tourism short video contents (information-oriented vs. emotion-oriented) on potential tourists’ travel intentions. Experiment 2 further explored how different tourism short video contents affect potential tourists’ travel intentions through the customer inspiration mechanism. Experiment 3 examined whether consumption orientation could moderate the relationship between different tourism short video content and potential tourists’ travel intentions.

This study has made three main contributions. Firstly, it enriched the mechanism of tourism short video content marketing from the perspective of customer inspiration. Secondly, this study examined the mediating mechanism and boundary conditions between different tourism short video contents and potential tourists’ travel intentions and illustrated the existence of a customer inspiration triggering mechanism of tourism short videos. Finally, this study provided suggestions for tourism managers in terms of further triggering customer inspiration by stimulating different consumption orientations and, in turn, designing more effective content marketing strategies for tourism short videos.

## 2. Literature review and hypothesis development

### 2.1. Content of short tourism videos

Tourism short videos are those in which tourists share their comments on the destinations, tourism products, and services they experienced using the destination as background and express their personal views on the experiences, services, or products (Perez-Vega et al., 2018; Liao et al., 2020). According to the previous video content analysis framework, tourism short videos can be roughly divided into two categories: information-oriented and emotion-oriented, which are the two main pathways to influence consumers’ attitudes (Maclnnis and Jaworski, 1989; Tellis et al., 2019).

Information-oriented short videos are designed to deliver information about product attributes, price information, service experiences, and brand events (Chandrasekaran et al., 2018). This type of video mainly satisfies the needs of tourists in the information-seeking stage with objective descriptions of destinations or products, the content of information is crucial in persuasion and communication (Hsieh et al., 2012). Tourists would consider information-oriented short video contents of greater reference value for traveling as long as the source is credible and the quality of information is high, and such positive inferences will increase destination information search and purchase intentions (Chandrasekaran et al., 2018; Munaro et al., 2021). Research has found that the attitudes elicited by information-oriented tourism short videos may vary depending on information presentation, content authenticity, and source credibility (Dedeoglu, 2019). For example, (Tellis et al., 2019) found information-oriented tourism short videos particularly dull and boring, even annoying. It shows that the results of existing studies on attitude change induced by information-oriented tourism short videos are not consistent, and further research is still needed.

Emotion-oriented short videos are designed to evoke emotional responses from the viewers using a variety of narrative storylines, including positive or negative emotions, and subsequently obtain a persuasive effect (Chandrasekaran et al., 2018; Tellis et al., 2019). Unlike the complex effects of information-oriented short videos, emotionally appealing videos are considered more persuasive (Nelson-Field et al., 2013). In fact, the emotional video contents (such as storylines, character settings, and music rhythms) trigger a variety of positive emotions in viewers (Liu et al., 2018), thus leading to more positive effects, such as longer viewing time (Teixeira et al., 2012), promoted content sharing (Tellis et al., 2019), and increased purchase intention (Lee and Hong, 2016). Compared to information-oriented short videos that focus on information content introducing new features of tourism products and new technology applications, the emotional content displayed in videos positively influences viewer behaviors.

When applying the above classification methods to tourism short videos, this study defines information-oriented tourism short videos as those focusing on tourist destination attraction information presentation and introduction of the historical background, cultural traditions, travel arrangements, price information, etc. of the destination. Emotion-oriented tourism short videos are defined as those showing the direct emotional connections between tourists and destinations in dramatic ways and drawing the viewers into the storytelling, thus activating their positive emotions. While both types of videos mentioned above are prevalent on social networking services (SNSs) such as YouTube and TikTok, in-depth analyses of how different tourism short video contents influence the attitudes and behaviors of potential tourists. To this end, this important topic is analyzed in this paper to fill the gaps in the existing literature.

### 2.2. Customer inspiration

Derived from the generalized concept of “inspiration” in social psychology (Thrash and Elliot, 2004), customer inspiration refers to a temporary activation state of the customer in a marketing situation, which promotes the shift from accepting marketing guidance and producing new ideas to the internal pursuit of consumption-related goals (Böttger et al., 2017). Customer inspiration consists of three stages: evocation, transcendence, and approach motivation, and is a second-order structural variable consisting of inspired-by and inspired-to. Inspired-by means that the customer receiving marketing guidance produces new ideas (i.e., evocation) and becomes aware of a new possibility that has never been discovered before (i.e., transcendence). Inspired-to means that the customer is eager to realize a new idea (e.g., to buy a product; Böttger et al., 2017). Many studies have confirmed that customer inspiration plays an important role in the travel information search stage and is of some value for predicting things like increased purchase intentions (Winterich et al., 2019), increased customer satisfaction (Winterich et al., 2019), and destination choice selection (Dai et al., 2022). For example, inspired by a friend’s health and travel photos on social media, a user may learn about a never considered wellness travel destination, which leads to a new travel idea to experience a landscape with healing effects. Driven by this motivation, the user may gradually realize the wellness travel dream by participating in health travel topic discussions, searching for additional travel information, and making travel plans (He et al., 2021; Dai et al., 2022).

According to the relevant literature, a large number of studies provided empirical evidence for the sources of customer inspiration and their triggering mechanisms in tourism short videos. Customer inspiration is not spontaneous but inspired by external marketing stimuli (Thrash and Elliot, 2004). Utilitarian and hedonistic content have also been found in some empirical studies to have a positive impact on customer inspiration (Izogo and Mpinganjira, 2020). For example, research has found that inspirational stimuli (print ads, novel product assortment, in-store demonstrations, personalized messages, consumption environments, etc.) and individual recipient characteristics (curiosity, nostalgia, openness, self-esteem, etc.) can influence the formation of customer inspiration, and customer inspiration have significant causal relationships with consumer attitudes, emotions, and behaviors (Böttger et al., 2017). In recent years, scholars have begun to introduce the concept of customer inspiration into the field of tourism. Hence, tourism inspiration, i.e., prompting potential tourists to transition from producing travel ideas upon tourism marketing stimuli to producing travel behaviors (Dai et al., 2022). However, this concept is only an extension of the customer inspiration concept. For example, (Khoi et al., 2020) found that place attachment and openness to experience had positive effects on tourist customer inspiration. Similarly, wellness tourism experiences can also evoke tourism inspiration in tourists, as the experience can bring intrinsic interest and motivation to the tourist to pursue the goal (He et al., 2021).

### 2.3. Content of tourism short videos and travel intention

Although studies have demonstrated the effects of tourism short video content on tourists’ behavioral intentions, questions about which content is more compelling need to be further explored in addition to further revealing the action mechanisms (Chi et al., 2022). Information-oriented is a more traditional way of content presentation in tourism short videos, which mainly contain tourism product attributes, price information, service experience, etc., and opinions are expressed by direct statements of facts and objective depiction (Chandrasekaran et al., 2018). Such short videos could bring high reference value to potential tourists in the planning stage as long as their contents are real and their pictures are clear, which grants them the effects of reducing risk, increasing trust, and promoting travel behavior intention (Tellis et al., 2019). Emotion-oriented is an emerging content presentation method in tourism short videos, especially on short video platforms such as YouTube and TikTok, which has received a lot of attention from researchers (Tellis et al., 2019; Gao et al., 2021; Chi et al., 2022). Research has found that emotion-oriented short videos are effectively edited for rhythm, sequence, and sound, which can control consumers’ emotional experience and increase their willingness to watch (Liu et al., 2018). Travel intention is often considered the attitude and likelihood of a potential tourist to visit a destination in the future and a prerequisite for tourist participation in tourism activities (Wang et al., 2022). Based on the customer inspiration theory, emotion-oriented tourism short videos take the destination as the scene, and the contents cover the storyline, character settings, and interesting expressions. The interesting and varied presentations of tourism experiences could promote new ideas for self-transcending tourism consumption, attract tourist attention, and stimulate their emotional responses (Gao et al., 2021; Dai et al., 2022; Xue et al., 2022). However, for information-oriented short videos, purely information-oriented contents are boring and uninteresting and may even irritate consumers (Tellis et al., 2019).

Based on the above discussion, the following hypotheses are formulated.

*H1*: Emotion-oriented tourism short video contents have a significant positive effect on potential tourists’ behavioral intentions compared to information-oriented tourism short video content.

### 2.4. Mediating effect of customer inspiration

Customer inspiration is a temporary motivation state produced by customers in response to marketing stimuli that leads consumers to produce new ideas and turn them into reality (Böttger et al., 2017). Customer inspiration is not spontaneously produced but inspired by external marketing stimuli (Thrash and Elliot, 2004). In terms of this study, the marketing stimulus is the tourism short video content. Tourism short video inspiration refers to the activation state of potential tourists from producing new ideas after receiving tourism marketing guidance to transforming ideas into the inner pursuit of tourism consumption-related goals, which is obtained from tourism short videos.

According to the customer inspiration theory, when potential tourists watch a tourism short video, its series of inspiring contents, such as destination scenery, destination activities and events, and fresh attempts in travel, may trigger them to form new ideas (inspire-by) about travel consumption with the spirit of self-transcendence. This temporary activation state further motivates potential tourists to urgently pursue tourism experience realization (inspire-to; Khoi et al., 2020). Thus, tourism short videos may be able to induce inspire-by and inspire-to. Since emotion-oriented tourism short videos are richer in content, more intense inspire-by and inspire-to can be induced under their stimulation.

Based on the above discussion, the following hypotheses are formulated.

*H2a*: Emotion-oriented tourism short video content can induce more intense inspired-by than information-oriented tourism short video content.

*H2b*: Emotion-oriented tourism short video content can induce more intense inspired-to than information-oriented tourism short video content.

Further, once a potential tourist is inspired by a marketing message such as a tourism short video (i.e., entering the inspired-by state), the potential tourist may discover a new and better possibility (which has never been discovered before) and form a sense of positivity, clarity, and self-improvement, thus producing an inner motivation to turn new ideas into action (Chi et al., 2022; Dai et al., 2022). At this time, a strong positive emotion often accompanies the inspiration, and research has found that customer inspiration itself has significant happy emotional arousal properties, such as pleasure (joy and excitement) and surprise. These positive emotions tend to trigger unplanned travel behavior intentions of potential tourists (Khoi et al., 2021; Dai et al., 2022). Therefore, we infer that emotion-oriented tourism short videos induce more intense customer inspiration in potential tourists under their marketing stimulation. This influences the travel intentions of potential tourists, i.e., customer inspiration mediates the positive relationship between tourism short video content and travel intentions.

Further, customer inspiration consists of two dimensions: inspire-by and inspire-to, which are in a coherent order (Xue et al., 2022). There is also a causal relationship between inspire-by and inspire-to, where inspire-by exists before inspire-to (Gao et al., 2021). For example, in a cross-cultural study (Izogo et al., 2020), found that the inspire-by state significantly and positively affected the inspire-to state. Similarly, research findings have been produced in studies on augmented reality (Hinsch et al., 2020), social media content (Izogo and Mpinganjira, 2020), and travel experience scenarios (Xue et al., 2022). Therefore, this study argues that the external marketing stimulus of tourism short videos first induces new ideas of tourism consumption (inspire-by) and then stimulates potential tourists to eagerly pursue the realization of tourism experience (inspire-to), thus promoting the formation of travel intentions.

On this basis, we formulate the following hypothesis.

*H3*: Compared with information-oriented tourism short video content, emotion-oriented tourism short video content positively influences potential tourists’ travel intentions through the chained mediation effect of inspire-by and inspire-to.

### 2.5. Moderating effect of consumption orientation

According to the existing research on customer inspiration, individual traits (such as curiosity, nostalgia, self-esteem, happiness, and motivation) affect inspiration generation by moderating the relationship between information sources and customer inspiration (Evanschitzky et al., 2014). Generally speaking, customers base their consumption activities on utilitarian or hedonistic motivations (Hirschman and Holbrook, 1982). Often associated with fun, frolic, enjoyment, and recreational experiences, hedonistic motivations seek subjective feelings such as emotions and personal symbols in the tourism experience. Utilitarian motivations, on the other hand, are goal-oriented rational behaviors focusing on objective factors such as the function, effect, and economic efficiency of the tourism process and tourism product (Babin et al., 1994; Shin and Jeong, 2021).

Research has found that compared to customers with utilitarian motivations, customers with hedonistic motivations are generally more daring and radical and more active in discovering new products, trying innovations, and accepting new things (Böttger et al., 2017). They also have a broader attention span and greater ability to capture information, which makes them more open-minded and creative (Friedman and Förster, 2001; Evanschitzky et al., 2014). Since individual open-mindedness and creativity are the main factors in the formation of inspiration, the possible correlation between hedonistic motivations and customer inspiration can be revealed (Böttger et al., 2017; Sheng et al., 2020). Thus, it can be inferred that when facing emotionally rich and experientially diverse emotion-oriented tourism short video content stimulation, tourists with hedonistic motivations produce more intense customer inspiration as they are more willing to accept new things, which, in turn, leads to stronger travel intentions during the travel information search stage. On the contrary, when facing the stimulus of information-oriented tourism short video content with detailed content and clear prices, tourists with utilitarian motivations may be more concerned about whether the short video content can meet their travel planning needs and thus less likely to produce stronger inspirations.

Therefore, we can formulate the following hypothesis.

*H4*: Consumption orientations moderated the chained mediation effect in which tourism short video contents affect the travel intentions through inspire-by and inspire-to, i.e., the chained mediation effect above is stronger for potential tourists holding hedonistic motivations than those holding utilitarian motivations.

### 2.6. Research framework

**Figure 1:**
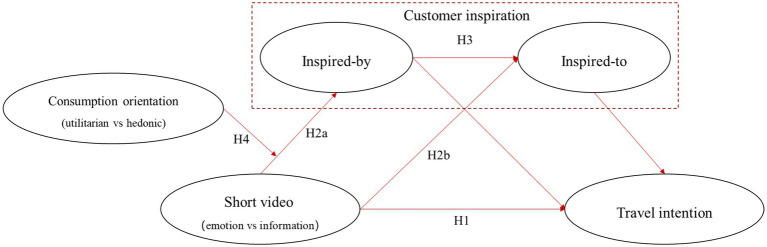
Research framework.

Based on the above hypotheses, as shown in Figure 1, tourism short video contents have a significant positive impact on travel intention (H1), and this relationship is mediated by customer inspiration (inspire-by and inspire-to; H3). Tourism short video contents have significant positive effects on inspire-by (H2a) and inspire-to (H2b), and consumption orientation moderates the chained mediation effect of tourism short video content through inspire-by and inspire-to (H4). The experimental method can exclude some confounding factors and more accurately infer the causal relationships between variables (Chi et al., 2022). Therefore, this study tested the above research hypotheses through three between-subjects experiments.

## 3. Experiment 1: Effect of short videos on travel intention

Experiment 1 aimed to explore the effects of different tourism short video contents on travel intentions. Since short videos are not professionally produced anytime and anywhere, and users focus on conveying information and emotional expression, studies have focused on the emotional and informational aspects of video content (Maclnnis and Jaworski, 1989; Aaker and Lee, 2001). Therefore, this study further subdivides short videos into information-oriented and emotion-oriented ones. A context-simulated single-factor (information-oriented and emotion-oriented) between-subjects experiment was used to test hypothesis 1.

### 3.1. Experimental design

#### 3.1.1. Participants

Experiment 1 was conducted at a university in China from October 12 to 17, 2021, on a paid (¥1 each) sample of 124 students with undergraduate and higher degrees from that university, including 62 in the information-oriented group and 62 in the emotion-oriented group. Male students accounted for 46.8% of the experimental sample, and female students accounted for 53.2%. The average age was21 years (*M* = 21.068, *SD* = 1.713). Subjects with daily viewing time lengths of 30 min or less, 30 to 60 min, 1 to 2 h, 2 to 4 h, and over 4 h accounted for 17.74, 22.58, 29.03, 24.19, and 6.45%, respectively.

#### 3.1.2. Materials

The research materials were selected from TikTok’s open videos based on the definition of tourism short videos in this paper. Considering the influence of subjects’ familiarity with the destination, the city of Qingdao, China, 1576.8 km away from the experimental site (Quanzhou, China) of this study, was selected as the tourist destination. As a famous tourist city in China with rich tourism resources and an important city for tourism short video promotion, Qingdao ranked 4th in terms of the comprehensive index in the March 2022 National Municipal Culture and Tourism New Media Communication Power Index Report released by the Culture and Tourism Industry Index Lab. The researchers searched the TikTok platform with the keyword “Qingdao tourism,” sorted the results by likes, and reviewed the top 500 Qingdao tourism short videos in detail. The tourism short videos were classified based on the existing research (Tellis et al., 2019), and the length (1 min), picture quality, and sound effects of the two classes of short videos were then standardized. The contents of information-oriented tourism short videos focus on the travel itinerary, including information on the itinerary, attractions, costs, and fun stuff about the trip to Qingdao. The content of emotion-oriented tourism short videos tells the story of a young woman who falls in love with a city because of a song in her pursuit of physical and mental freedom and the footage of her chasing the scenery in the city.

Through the pilot study, we performed a manipulation check to verify the effectiveness of short video stimuli. Specifically, 74 subjects were recruited through payment (¥1) and randomly assigned into two groups to watch one of the videos. After watching the video, subjects filled out a questionnaire to determine the type of video content (information-oriented or emotion-oriented) first and then to distinguish (1) to what extent did the video focus on presenting information about the destination and (2) to what extent did the video focus on appealing to your emotions (1 = very weak, 7 = very strong; Tellis et al., 2019). Pilot study manipulation check results and independent samples *t*-test showed a significant difference between information-oriented and emotion-oriented short videos (*t* = 6.750, *df* = 72, *p* < 0.000). For screening question 1, the scores of the information-oriented group were significantly higher than the emotion-oriented group (*M*_inf_ = 5.189, *M*_emo_ = 4.243, *t* = 3.515, *p* = 0.001). For screening question 2, the scores of the information-oriented group were significantly lower than the emotion-oriented group (*M*_inf_ = 3.946, *M*_emo_ = 4.892, *t* = −3.735, *p* < 0.000). Taken together, the short video experiment material was successful and effective in manipulating the independent variables, and thus a formal experimental study could be conducted on top of this.

#### 3.1.3. Procedure

The complete experimental procedure consists of three parts. First, the invited subjects were randomly assigned to two groups and briefed on the experimental requirements and procedure. Then, the experimental assistant randomly selected one of the two videos and presented it. Finally, the experimental assistant distributed questionnaires to the subjects, asking them to judge the type of the video and report their travel intentions to the destination, their familiarity with the destination in the video, their familiarity with the characters in the video, and their demographic information.

The travel intention scale was acquired from relevant literature (An et al., 2021; Chi et al., 2022), where all items were measured on a 7-point Likert scale (1 = extremely disagree, 7 = extremely agree) and was translated strictly according to the “English to Chinese” first and then “Chinese to English” procedure.

### 3.2. Results

#### 3.2.1. Manipulation check

According to the independent samples *t*-tests, the two groups of subjects were able to clearly distinguish between information-oriented and emotion-oriented short videos (*t* = 9.763, *df* = 122, *p* < 0.000), their familiarity with the destination was not significantly different (*M*_inf_ = 2.613, *M*_emo_ = 2.484, *t* = 0.897, *p* = 0.371), neither was their familiarity with the character (*M*_inf_ = 2.161, *M*_emo_ = 2.032, *t* = 0.717, *p* = 0.474). Therefore, the experimental manipulation was successful.

#### 3.2.2. Test of the main effect

In this study, one-way variance analysis (ANOVA) was conducted to test the differences in the effects of different types of short videos on tourists’ travel intentions. Data analysis results showed that the different types of short videos had significantly different effects [*M*_inf_ = 3.887, *M*_emo_ = 4.919, *F*(1, 122) = 26.547, *p* < 0.000] on travel intentions (*α* = 0.804), indicating that emotion-oriented short videos were more likely to inspire tourists’ travel intentions than information-oriented short videos, thus, validating H1.

## 4. Experiment 2: Mediating effect of customer inspiration

Experiment 1 examined the main effects of different tourism short video contents on potential tourists’ travel intentions. To further explore the possible mediating mechanisms involved, Experiment 2 was conducted on top of this. Experiment 2 further explored how different tourism short video contents affect potential tourists’ travel intentions. A context-simulated single-factor (information-oriented and emotion-oriented) between-subjects experiment was also conducted in Experiment 2, and the effects of different types of short videos on customer inspiration and the mediating effect of customer inspiration on the relationship between different short videos and travel intentions were tested to verify H2a, H2b, and H3.

### 4.1. Experimental design

#### 4.1.1. Participants

Experiment 2 was conducted from December 10 to 15, 2021, on 216 university students recruited by payment (¥1 each) from a university in southern China. Of those, 51.4% were male, and the average age was 20 years (*M* = 20.120, *SD* = 1.402). Subjects with daily viewing time lengths of 30 min or less, 30 to 60 min, 1 to 2 h, 2 to 4 h, and over 4 h accounted for 24.07, 27.78, 33.80, 12.04, and 2.31%, respectively.

#### 4.1.2. Procedure

The same video used in Experiment 1 was used as the stimulus in Experiment 2, and a data collection procedure similar to that in Experiment 1 was used. All subjects were randomly assigned to two groups to watch either the information-oriented short video (n = 107) or the emotion-oriented short video (n = 109). After viewing, subjects were asked to judge the type of the video and complete the customer inspiration and travel intention measurement scales (Böttger et al., 2017; An et al., 2021; Chi et al., 2022; see Appendix A for specific scale items) and report their familiarity with the destination and characters in the video and their basic demographic information.

### 4.2. Results

#### 4.2.1. Manipulation check

According to the independent samples *t*-tests, the two groups of subjects were able to clearly distinguish between information-oriented and emotion-oriented short videos (*t* = 22.913, *df* = 214, *p* < 0.000), their familiarity with the destination was not significantly different (*M*_inf_ = 2.785, *M*_emo_ = 2.908, *t* = 0.958, *p* = 0.339), neither was their familiarity with the character (*M*_inf_ = 2.000, *M*_emo_ = 2.119, *t* = 0.922, *p* = 0.358). Therefore, the experimental manipulation was successful.

#### 4.2.2. Main effect test and chained mediation effect test

In this study, one-way variance analysis (ANOVA) was conducted to test the differences in the effects of different types of short videos on tourists’ travel intentions. Data analysis results showed that the different types of short videos (0 = information-oriented, 1 = emotion-oriented) had significantly different effects [*M*_inf_ = 3.266, *M*_emo_ = 4.438, *F*(1, 245) = 26.021, *p* < 0.000] on travel intentions (*α* = 0.804), indicating that emotion-oriented short videos were more likely to inspire tourists’ travel intentions than information-oriented short videos, thus, again validating H1.

In this study, one-way variance analysis (ANOVA) was conducted to test the differences in the effects of inspired-by and inspired-to on tourists’ travel intentions. According to the results, the different types of short videos had significantly different effects [*M*_inf_ = 3.843, *M*_emo_ = 4.624, *F*(1, 245) = 20.856, *p* < 0.000] on inspired-by customer inspirations (α = 0.859), thus validating H2a; the different types of short videos had significantly different effects [*M*_inf_ = 3.944, *M*_emo_ = 5.121, *F*(1, 245) = 34.368, *p* < 0.000] on inspired-to customer inspirations (α = 0.928), thus validating H2b.

**Figure 2:**
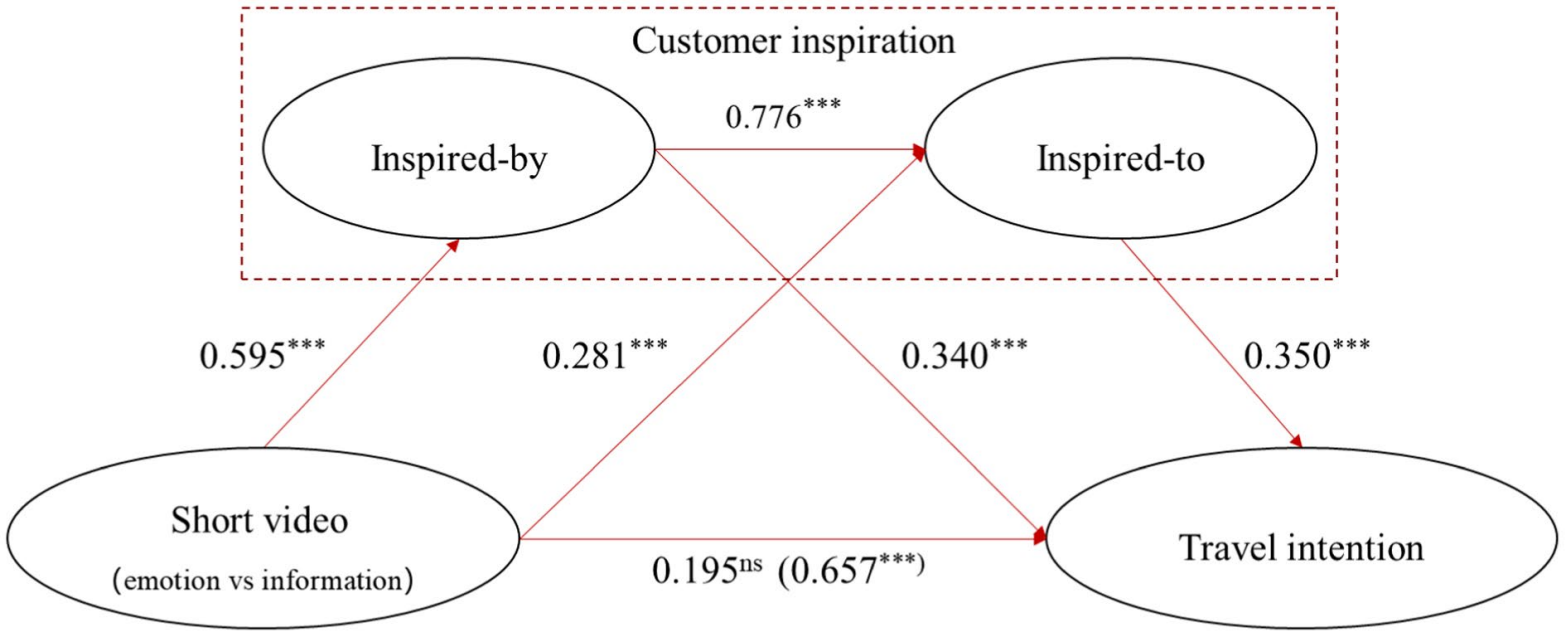
The continuous mediation effects of inspired-by and inspired-to customer inspirations in the effect of short videos on travel intentions.***Represents a significant level of 1%.

To investigate the chain mediation effect of inspired-by customer inspiration and inspired-to customer inspiration, this study used Model 6 of the PROCESS plugin provided by Hayes and Rockwood (2017) for bootstrapping path analysis, where the number of repeat measurement samples was set to 5,000, and the confidence interval was set to 95%. Different short video contents (0 = information-oriented, 1 = emotion-oriented) were used as independent variables, travel intention was used as the dependent variable, and inspired-by customer inspiration and inspired-to customer inspiration were used as chained mediation variables. The entire regression equation was significant, with *R^2^* = 0.108, *F*(1, 214) = 26.021, and *p* < 0.000. The path coefficient results are shown in Figure 2. The results in Figure 2 show that, compared with information-oriented short videos, emotion-oriented short videos have a significant and positive impact on inspired-by customer inspiration [*β* = 0.595, *t*(1, 214) = 4.567, *p* < 0.000]. The emotion-oriented short videos have a significant and positive impact on inspired-to customer inspiration [*β* = 0.281, *t*(2, 213) = 3.505, *p* < 0.000]. Inspired-by customer inspirations have a significant and positive impact on inspired-to customer inspirations [*β* = 0.766, *t*(2, 213) = 19.331, *p* < 0.000]. Inspired-by customer inspirations have a significant and positive impact on travel intentions [*β* = 0.340, *t*(3, 212) = 3.980, *p* < 0.000]. Inspired-to customer inspirations have a significant and positive impact on travel intentions [*β* = 0.350, *t*(3, 212) = 3.978, *p* < 0.001]. After adding continuous mediating variables, the regression coefficient [*β* = 0.657, *t*(1, 214) = 5.101, *p* < 0.000] of emotion-oriented short videos on travel intention is no longer significant [*β* = 0.195, *t*(3, 212) = 1.846, *p* = 0.066] compared to information-oriented short videos. In terms of the three mediation paths, the “short video – inspired-by customer inspiration – travel intention” path is significant [95% CI = (0.075, 0.352), not including zero], the “short video – inspired-to customer inspiration – travel intention” path is significant [95% CI = (0.031, 0.188), not including zero], and the “short video – inspired-by customer inspiration – inspired-to customer inspiration – travel intention” path is significant [95% CI = (0.062, 0.284), not including zero]. The above results validate H3.

Thus, it is concluded that emotion-oriented tourism short videos (vs. information-oriented ones) increase inspired-by customer inspirations, which, in turn, leads to higher inspired-to customer inspirations and finally strengthens the travel intentions, thus proving H3.

## 5. Experiment 3: Moderating effect of consumption orientation

Experiment 2 verified that mediating effect of customer inspiration in the relationship between short videos and travel intentions. Experiment 3 examined whether tourist consumption orientations had a moderating effect in that relationship, where a 2 (information-oriented vs. emotion-oriented short videos) × 2 (utilitarian vs. hedonistic consumption orientations) matrix was used to validate H4.

### 5.1. Experimental design

#### 5.1.1. Participants

Experiment 3 was conducted from March 11 to 15 and May 20, 2022, on 236 undergraduates recruited by payment (¥2) from three universities in southern China. Of those, 34.3% were male, and the average age was 20 years (*M* = 20.120, *SD* = 1.402). Subjects with daily viewing time lengths of 30 min or less, 30 to 60 min, 1 to 2 h, 2 to 4 h, and over 4 h accounted for 20.34, 33.05, 33.05, 11.44, and 2.12%, respectively. Questionnaire respondents were recruited by payment, and no duplicate responses or inattentive responses were found.

#### 5.1.2. Procedure

The same video used in Experiment 1 was used as the stimulus in Experiment 3, and a data collection procedure similar to that in Experiment 1 was used. All subjects were randomly assigned to four groups to watch utilitarian and information-oriented short videos (*n* = 60), utilitarian and emotion-oriented short videos (*n* = 66), hedonistic and information-oriented short videos (*n* = 54), and hedonistic and emotion-oriented short videos (*n* = 56). First, the subjects were told that they were about to prepare for a trip. Specifically, the utilitarian group was told that the aim of the trip was to save as much money as possible while visiting as many sites as possible. The hedonistic group was told that the aim of the trip was to enjoy as much freedom of mind and body as possible. After that, the experimental assistant began to play the short videos. After viewing, the subjects were asked to report their travel motivations (Screening Question 1, the trip was less costly; Screening Question 2, the trip was in search of freedom and pleasure), judge the type of the short video content, complete the customer inspiration and travel intention measurement scales (Böttger et al., 2017; An et al., 2021; Chi et al., 2022), and report their familiarity with the destination and characters in the video and their basic demographic information.

### 5.2. Results

#### 5.2.1. Manipulation check

The independent samples *t*-test results revealed that the two groups of subjects were able to clearly distinguish between the information-oriented and emotion-oriented short videos (*t* = 19.211, *df* = 234, *p* < 0.000). In terms of different travel motivations, the two groups of subjects differed in the purpose of spending less (*M*_uti_ = 4.921, *M*_hed_ = 4.073, *t* = 4.460, *p* < 0.000) and the purpose of finding freedom and pleasure (*M*_uti_ = 4.524, *M*_hed_ = 5.955, *t* = −11.407, *p* < 0.000). The two groups had no difference in familiarity with the destination [*F*(1, 235) = 1.041, *p* = 0.309] and the character [*F*(1, 235) = 1.482, *p* = 0.225]. Therefore, the experimental manipulation was successful.

#### 5.2.2. Moderating effect test

In this study, one-way variance analysis (ANOVA) was conducted to test the differences in the effects of different short video contents on potential tourists’ travel intentions. The results showed that the different short video contents had significantly different effects [*M*_inf_ = 3.974, *M*_emo_ = 4.457, *F*(1, 235) = 11.237, *p* = 0.001] on travel intentions (*α* = 0.813), indicating that emotion-oriented short videos were more likely to inspire tourists’ travel intentions than information-oriented short videos, thus, again validating H1.

In this study, one-way variance analysis (ANOVA) was conducted to test the differences in the effects of inspired-by and inspired-to on tourists’ travel intentions. According to the results, the different types of short videos had significantly different effects [*M*_inf_ = 4.105, *M*_emo_ = 4.729, *F*(1, 235) = 30.066, *p* < 0.000] on inspired-by customer inspirations (α = 0.829), thus validating H2a; the different types of short videos had significantly different effects [*M*_inf_ = 4.091, *M*_emo_ = 4.944, *F*(1, 235) = 37.150, *p* < 0.000] on inspired-to customer inspirations (α = 0.902), thus validating H2b.

Hypothesis 3 predicts the chained mediation effect of inspired-by customer inspiration and inspired-to customer inspiration. This study used Model 6 of the PROCESS plugin provided by Hayes and Rockwood (2017) for bootstrapping path analysis, where the number of repeat measurement samples was set to 5,000, and the confidence interval was set to 95%. Different short video contents (0 = information-oriented, 1 = emotion-oriented) were used as independent variables, travel intention was used as the dependent variable, and inspired-by customer inspiration and inspired-to customer inspiration were used as chained mediation variables. The entire regression equation was significant, with *R^2^* = 0.046, *F*(1, 234) = 11.237, and *p* < 0.000. The mediation effect test results are shown in Table 1. The results indicate that the direct effect is-0.102 (95% CI = [−0.343, 0.139]), the “short video – inspired-by customer inspiration – travel intention” path is significant [95% CI = (0.097, 0.327), not including zero], the “short video – inspired-to customer inspiration – travel intention” path is significant [95% CI = (0.062, 0.306), not including zero], the “short video – inspired-by customer inspiration – inspired-to customer inspiration – travel intention” path is significant [95% CI = (0.113, 0.349), not including zero], and all indirect effect is 0.585 (95% CI = [0.373, 0.825]). Consistent with the results of Experiment 2, the chained mediation effects of inspired-by customer inspiration and inspired-to customer inspiration are valid, and the above results validate H3.

**Table 1:** The continuous mediation effects of inspired-by and inspired-to customer inspirations in the effect of short videos on travel intentions.

Paths	Estimations	*SE*	95% Confidence interval	
			LLCI	ULCI
Short video – inspired-by customer inspiration – travel intention	0.198	0.059	0.097	0.327
Short video – inspired-to customer inspiration – travel intention	0.171	0.062	0.062	0.306
Short video – inspired-by customer inspiration – inspired-to customer inspiration – travel intention	0.216	0.060	0.113	0.349
Total indirect effect	0.585	0.116	0.373	0.825
Direct effect	-0.102	0.122	−0.343	0.139
Total effect	0.483	0.144	0.199	0.767

To test hypothesis 4, we imported the independent variable of different types of short video content (0 = information-oriented, 1 = emotion-oriented), the dependent variable of travel intention, the mediating variables of inspired-by and inspired-to customer inspirations, and the moderating variable of travel motivation into the Model83 in the PROCESS plugin provided by Hayes and Rockwood (2017), and the results are shown in Table 2. Under utilitarian travel motivations, the continuous mediation effects of inspired-by and inspired-to customer inspirations between short videos and travel motivations were not valid, *β* = *0*.094, SE = 0.057, 95% confidence interval [−0.005, 0.219]. Under hedonistic travel motivations, the continuous mediation effects of inspired-by and inspired-to customer inspirations between short videos and travel motivations were not valid, *β* = *0*.359, SE = 0.088, 95% confidence interval [0.201, 0.546]. Thus, the chained mediation effects under different travel motivations differed significantly, *β*_hed-uti_ = 0.265, SE = 0.087, 95% confidence interval [0.105, 0.449]. Therefore, H4 was verified, i.e., travel motivation moderated the chained mediation effects of tourism short video content on travel intentions through inspire-by and inspire-to.

**Table 2:** Comparison of continuous mediation effects in different scenarios under the moderation of travel motivation.

Travel motivations	Estimations	*SE*	95% Confidence interval	
			LLCI	ULCI
Utilitarian travel motivation	0.094	0.057	−0.005	0.219
Hedonistic travel motivation	0.359	0.088	0.201	0.546
Diff (hedonistic-utilitarian)	0.265	0.087	0.105	0.449

## 6. Conclusion and discussion

### 6.1. Conclusion

In this study, three experiments were conducted to explore the impact of different types of tourism short video content on potential tourists’ travel intentions. We came to the following conclusions. Firstly, different tourism short video contents significantly affect potential tourists’ travel intentions. The results show that emotion-oriented short videos are more likely to stimulate potential tourists’ travel intentions than information-oriented short videos. Emotion-oriented short videos are conducive to facilitating potential tourists to make travel decisions. Secondly, the results show that emotion-oriented short videos induce stronger inspired-by and inspired-to customer inspirations than information-oriented short videos, prompting potential tourists to form stronger customer inspirations. Under the chained mediation effects of inspired-by and inspired-to inspirations, the direct effect of emotion-oriented short videos on potential tourists’ travel intentions is no longer significant, but the chained mediation effect is significant. These results suggest that emotion-oriented short videos indirectly increase potential tourists’ travel intentions by stimulating their customer inspiration. Finally, consumption orientation moderates the chained mediation effects of tourism short video content on travel intentions through inspire-by and inspire-to; For potential tourists holding hedonistic motivations, tourism short videos induce stronger new ideas of travel experiences in them, which, in turn, create the urge to acquire such tourism experiences, eventually reinforcing the travel intentions in the tourism decision-making process. In contrast, for customers with utilitarian motivations, inspire-by and inspire-to have no significant effect on the chain mediating effect between short videos and travel intention. The above study expands the perspective of tourism short video research (Cao et al., 2021; Chi et al., 2022; Dai et al., 2022) and enriches the existing literature by illustrating how tourism short videos enhance travel intentions by improving customer inspiration. In addition, this study further extends the existing literature by confirming the mediating effect of customer inspiration in the relationship where tourism short videos drive potential tourists’ travel intentions.

### 6.2. Theoretical contribution

As one of the few studies on tourism short videos from a customer inspiration perspective, this paper makes the following important theoretical contributions.

Firstly, this study provides a promising new perspective for tourism short video research. The existing research on tourism short videos mainly focuses on destination short video marketing (Wengel et al., 2022), tourism short video dissemination (Cao et al., 2021; Wang et al., 2022), tourism short video interaction (Xu et al., 2021), etc. However, key questions about the behavior of tourism short video users have not yet received sufficient attention. A few studies have focused on the impact of tourism short videos on the behavior of potential tourists, and the intrinsic mechanisms were explained from the perspectives of social capital (Liao et al., 2020), familiarity (Xue et al., 2022), brand (Chi et al., 2022), etc. However, those studies did not explain the differences in the action mechanisms of different tourism short video contents, and the mechanism of tourism short video user behaviors requires further exploration. Unlike previous studies, this study provides a new perspective to the existing literature on the behaviors of tourism short video users. From the customer inspiration perspective, this study points out that the emotionally rich storytelling emotion-oriented tourism short videos (vs. information-oriented ones) are helpful in increasing potential tourists’ travel intentions, which enriches the previous theoretical framework on the behaviors of tourism short video users.

Secondly, this study applies the customer inspiration theory to further explore the intrinsic mechanism by which tourism short videos drive potential tourists’ travel intentions and successfully verifies the chained mediation effects of inspire-by and inspire-to in that mechanism, which enriches the research on the intrinsic mechanism of tourism short videos. Customer inspiration theory suggests that inspiration is a mediating relationship between external marketing stimuli and behavioral intentions. Based on customer inspiration theory, this study proposes a new mechanism driving the formation of potential tourists’ travel intentions:emotion-oriented tourism short videos (vs. information-oriented ones) with rich emotions and storytelling narratives promote potential tourists’ travel intentions by stimulating their customer inspirations (including the generation of new ideas and the urge to acquire travel experiences). The results of this empirical study validate the hypotheses proposed based on the framework of customer inspiration theory and demonstrate the applicability of customer inspiration theory in explaining the behavior mechanism of tourism short video users, which contributes to the application of customer inspiration theory in the field of tourism short video content marketing.

Finally, this study explores the factors influencing potential tourists’ travel intention and uses consumption orientation as a moderating variable, which helps to expand the boundary conditions for the positive impact of tourism short videos. Previous studies have also found that among different consumption orientations, hedonistic motivations promote the formation of customers’ behavioral intentions (Pestana et al., 2020; Wang et al., 2022). This study obtains a similar finding to previous studies. Experiment 3 shows that the chained mediation effect of tourism short videos influencing intention to travel through inspire-by and inspire-to holds for potential travelers with hedonistic motivations but not for potential travelers with utilitarian motivations. Therefore, exploring the potential effects of consumption orientations on the relationship between different tourism short video contents and travel intentions is of great significance to the formation mechanism of potential tourists’ travel intentions.

### 6.3. Practical implications

In addition to theoretical contributions, this study also provides some suggestions on tourism short video content marketing.

Firstly, the findings in this study confirm that tourism short videos, especially emotion-oriented tourism short videos, have a facilitating effect on travel intentions from the customer inspiration perspective. On the one hand, the increasing number of entities providing tourism short video content must provide higher quality content in order to stand out from the large number of short videos and acquire the limited attention resources of potential tourists. On the other hand, high-quality tourism short video content marketing must stimulate potential tourists’ customer inspirations and effectively transform the short video content and tourism products. Thus, tourism short video content must be designed from three aspects: tourism product perspective, tourist perspective, and short video content perspective.

Secondly, the findings in this study point out that customer inspiration is an important mediating variable through which tourism short videos affect potential tourists’ travel intentions. Therefore, tourism enterprise managers should first clearly understand that stimulating customer inspiration is not easy and draw the power of tourists as mobile self-media carriers by providing a large number of short video materials to facilitate their creation; For consumers, customer inspiration may stimulate the potential traveling intention. However, constraints such as travel, accommodation, and economy must be considered to reduce the failure of the tourism experience caused by impulsive decision-making. Secondly, flexible editing and choreography skills are also important ways of inspiration. Tourism agency managers can make a large number of skill templates through platforms such as TikTok for direct use by short video creators, thus promoting tourism marketing of the destination. Thirdly, tourism agency managers should further explore the cultural connotation and shape tourism branding IPs to reduce the time for tourists to produce inspiration and compress the possibility of ambiguity in the tourism decision-making process.

Finally, the results showed that hedonistic motivations had a stronger impact on potential tourists’ customer inspiration. Therefore, tourism agencies should first use digital means to identify different consumer motivations using “baits” such as most beautiful attraction contests, thus segmenting potential tourists from the motivation perspective. Secondly, emotion-oriented tourism short videos should be distributed to groups with hedonistic motivations to stimulate their customer inspirations. In addition, tasks such as sharing, liking, and commenting should be assigned to enhance their stickiness with tourism agencies, including the destination and scenic spot. Detailed tour information about the destination should be distributed to groups with utilitarian motivations to save the time cost.

### 6.4. Limitations and future research

Although this study expands the perspective to explain the relationship between tourism short videos and travel intentions, some limitations remain.

Firstly, we conducted three experiments and used three different Chinese samples to test the hypotheses, which ensured the preciseness of the experimental method but limited the generalizability of the findings as these data were collected in China. Therefore, future studies may examine the relationship between the variables with a larger and more diverse population and scales of good reliability from a cross-cultural perspective.

Secondly, although this study used real short videos as experimental materials in the scenario-based experiments, the subjects’ contextual integration and emotional experience may not be as authentic. Therefore, future research can investigate the relationship between different types of tourism short videos and travel intention *in situ*. Cooperation with tourism short video platforms (such as YouTube and TikTok) can also be considered to analyze the relationship between tourism short videos and tourism consumption in different tourism consumption stages using the real consumption data available on those platforms, thus further expanding this study.

Thirdly, using questionnaire scales to measure customer inspiration, a psychological state, might lead to a certain subjective bias. In the future, a more accurate multi-channel physiological instrument could be used to obtain objective data for more precise impact effect analysis in real situations.

Finally, only short video content was selected as an influencing factor on the travel intention in this study, while other factors may also affect potential tourists’ travel intentions, such as short video characters and short video usage experience. In the future, subsequent studies can further explore the mechanism by which tourism short videos promote travel intention formation and short video content marketing strategies through in-depth interviews and focus groups, thus further improving our study on this basis.

## Data availability statement

The original contributions presented in the study are included in the article/Supplementary material, further inquiries can be directed to the corresponding author.

## Author contributions

GW is responsible for the research design, data collection, draft writing, and paper revision of this project. XD was responsible for part of the literature collection. All authors contributed to the article and approved the submitted version.

## Conflict of interest

The authors declare that the research was conducted in the absence of any commercial or financial relationships that could be construed as a potential conflict of interest.

## Publisher’s note

All claims expressed in this article are solely those of the authors and do not necessarily represent those of their affiliated organizations, or those of the publisher, the editors and the reviewers. Any product that may be evaluated in this article, or claim that may be made by its manufacturer, is not guaranteed or endorsed by the publisher.
