# Supplementary material for: Which type of tourism short video content inspires potential tourists to travel
Source: Front Psychol. 2023 Mar 1;14:1086516. doi: 10.3389/fpsyg.2023.1086516 (PMC10014723; doi:10.3389/fpsyg.2023.1086516)
Supplement: Supplementary file 1 [file Table_1.DOCX]

Appendix A: measurement scale

| Factors | Items | extremely disagree | strongly disagree | disagree | neutral | agree | strongly agree | extremely agree |
| --- | --- | --- | --- | --- | --- | --- | --- | --- |
| Inspired-by | Because of the content of this short video, | | | | | | | |
|  | my imagination was stimulated. | 1 | 2 | 3 | 4 | 5 | 6 | 7 |
|  | I was intrigued by a new idea. | 1 | 2 | 3 | 4 | 5 | 6 | 7 |
|  | I unexpectedly and spontaneously got new ideas. | 1 | 2 | 3 | 4 | 5 | 6 | 7 |
|  | my horizon was broadened. | 1 | 2 | 3 | 4 | 5 | 6 | 7 |
|  | I discovered something new. | 1 | 2 | 3 | 4 | 5 | 6 | 7 |
| Inspired-to | Because of the content of this short video, | | | | | | | |
|  | I was inspired to buy something. | 1 | 2 | 3 | 4 | 5 | 6 | 7 |
|  | I felt a desire to buy something. | 1 | 2 | 3 | 4 | 5 | 6 | 7 |
|  | my interest to buy something was increased. | 1 | 2 | 3 | 4 | 5 | 6 | 7 |
|  | I was motivated to buy something. | 1 | 2 | 3 | 4 | 5 | 6 | 7 |
|  | I felt an urge to buy something. | 1 | 2 | 3 | 4 | 5 | 6 | 7 |
| Travel intention | I plan to visit places that appeared in the video in the near future. | 1 | 2 | 3 | 4 | 5 | 6 | 7 |
|  | I will make an effort to visit places that appeared in the video in the near future. | 1 | 2 | 3 | 4 | 5 | 6 | 7 |
|  | I have the intention to visit places that appeared in the video in the near future. | 1 | 2 | 3 | 4 | 5 | 6 | 7 |
|  | I am willing to visit places that appeared in the video in the near future. | 1 | 2 | 3 | 4 | 5 | 6 | 7 |
